# Supplementary material for: MGEnrichment: A web application for microglia gene list enrichment analysis
Source: PLoS Comput Biol. 2021 Nov 17;17(11):e1009160. doi: 10.1371/journal.pcbi.1009160 (PMC8598070; doi:10.1371/journal.pcbi.1009160)
Supplement: Attachment — Submitted filename: ResponsetoReviews.pdf [file pcbi.1009160.s003.pdf]

We would like to thank the reviewers for their insightful feedback on our work. We appreciate the comments that the *release of MGEnrichment is timely and will likely facilitate data analysis and interpretation. Based on my testing, it is quite user-friendly, and results are quickly generated* (reviewer 1) and that *we found the article and web tool of high quality and of appropriate scope for PLoS Comp Bio* (reviewer 2). We also note that reviewer 2 and their lab *were able to adequately test the tool and found its functionality helpful in interpreting microglial gene sets that we had generated in our lab,* which was the exact functionally we were targeting with our tool.

Both reviewers requested the addition of a human version of the database, the inclusion of several additional datasets and several aesthetic changes to the output files produced by MGEnrichment. We have addressed all of these requests as detailed below and feel that these changes have significantly improved our tool and the accompanying manuscript for publication in PLOS Comp Bio.

Reviewer #1: …it is a little perplexing to see that recent seminal work on human microglia were not included in the first iteration of the software. For example, work from Bart Eggen's lab (PMID 28671693 and 32732419) and Chris Glass' lab (e.g., PMID 28546318) have provided high-quality, comprehensive datasets of microglial gene expression from human microglia, and these are frequently used as baseline to generate and refine protocols for the generation of ips-derived microglia….I would be inclined to ask that the authors add a human component, as the resulting enhanced scope would be better aligned with the goal of PLOS Computational Biology.

We greatly appreciate the suggestion of inclusion of a human version of the application and have now included the option for users to input either human or mouse genes and conduct enrichment analysis against either a human or mouse version of the database. To construct the human database, we went back to the original papers and re-pulled the corresponding gene lists so that the human database includes all genes identified in the original publications (not just those that have homologous mouse genes in the mouse database). In addition, we added in the three human datasets noted by the reviewer as well as several other human and mouse datasets to bring the total number of gene lists in the databases to 214 (previously 166).

Currently, the mouse database contains mouse gene ids from mouse, rat and human with the rat and human converted to mouse using biomart. The human database contains human gene ids from human, rat and mouse with rat and mouse being converted to human using biomart. In addition to the updated database, we updated the corresponding summary files (supplemental table 1), documentation and the instructions on our github page for how to add new mouse or human genes to the two databases. This includes instructions on how to perform the conversion from mouse to human or human to mouse using biomart.

Reviewer #2: Summary of MGEnrichment: a web application for microglia gene list enrichment analysis

We had a few minor comments with the manuscript / list of datasets with microglial datasets:
• It'd be helpful to include a table in the manuscript listing the datasets and publications used

We have now included this as supplemental table 1 that includes information on both the mouse and human databases.

• How carefully was the literature curated, in particular, the list of human microglial subtypes? We found the omission of the datasets presented in Olah et al., 2020 as one such missing dataset we had expected to be included: https://www.nature.com/articles/s41467-020-19737-2

We apologize for this oversight as we were previously unaware of this paper. Both the microglial cluster genes and state genes have been added to both the mouse and human databases.

In using the associated web application, we noticed a few points for improvement that would greatly increase the usability of the application.

• Given that our gene lists were based on analyses of human cells, we found it inconvenient to have to first convert these genes to the equivalent gene names in the mouse. This seemed non-intuitive and unnecessary, especially as a number of the gene lists are based on human gene expression profiles. I suggest adding functionality to allow the user to enter what species their gene names are entered as, and do the conversion of the symbols within the app.

We have now expanded the app to include the option of uploading either a mouse or human set of gene IDs and to compare those IDs against either a mouse or human database.

• The gene list groups that are selected by default are non-intuitive. Given that this is a microglia enrichment tool, it seems more useful and intuitive to have only the microglia, microglia development, and inflammation gene list groups selected by default.

We appreciate this feedback, and have limited the default selected lists to be "microglia", "microglia development", and "inflammation", as suggested.

• It'd be more useful to pick a more meaningful default for the minimum FDR value – I'd suggest a value of 0.1 instead of 1.0.

We appreciate this suggestion, and have modified the default FDR value to be 0.05, in accordance with the FDR value filtering most commonly used for this type of analysis.

• *The notAnotB inAnotB terminology is not intuitive. Consider using the terms N K n k from hypergeometric test:* [*https://en.wikipedia.org/wiki/Hypergeometric_distribution*](https://en.wikipedia.org/wiki/Hypergeometric_distribution)

We appreciate the feedback about the confusing nature of the previous terminology. To make the column names more intuitive for novice users who may not be familiar with statistical notation, we have changed the column names to specify the number of genes that are in the user list only, the database only, in both lists, or in neither list.

• *There are a lot of columns displayed within the tool – are all of these really necessary? I don't find it particularly helpful to show both the intersection IDs, intersection ensembl, intersection mgi symbol, etc etc. I think merely showing the gene symbols would be more than sufficient. Showing all of these columns makes each row within the app very long and challenging to scroll through.*

We appreciate the feedback about the clarity of the user interface, and have changed the application to show only gene symbols by default.

• *The sorting of results that are displayed in the table would be considerably more intuitive if the table was sorted by default in order of increasing pvalue or FDR.*

We have modified the table to be sorted by increasing values of the FDR column.

• *Is it possible to have the source publications be listed as hyperlinks with links to the publications at pubmed?*

We have added a new column to the database "full.source" that includes the full paper citation including a doi link to each publication.Doi links were chosen as they are the most stable web linkage to publications.

• *Please think carefully about where columns in the table should appear. For example, I found the shortnames for each condition somewhat difficult to parse. The problem would likely be fixed if the columns listname, shortname, and description were closer to one another (also – why do you need all 3).*

We appreciate this comment and have removed the "shortname" description as we agree it was redundant with the listname.
